# LGR5 promotes the proliferation and tumor formation of cervical cancer cells through the Wnt/β-catenin signaling pathway

**DOI:** 10.18632/oncotarget.2377

**Published:** 2014-08-21

**Authors:** Qing Chen, Hao-Zhe Cao, Peng-Sheng Zheng

**Affiliations:** ^1^ Department of Reproductive Medicine, the First Affiliated Hospital, Xi'an Jiaotong University Medical School, Xi'an, the People's Republic of China; ^2^ Department of Biochemistry and Molecular Biology, Xi'an Jiaotong University Medical School, Xi'an, the People's Republic of China; ^3^ Division of Cancer Stem Cell Research, Key Laboratory of Environment and Genes Related to Diseases, Ministry of Education, Xi'an Jiaotong University Medical School, Xi'an, the People's Republic of China

**Keywords:** LGR5, cervical cancer, proliferation, cell cycle, Wnt/β-catenin signaling

## Abstract

Leucine-rich repeat-containing G protein-coupled receptor 5 (LGR5), a seven transmembrane receptor known as a potential stem cell marker for intestinal crypts and hair follicles, has recently been found to be overexpressed in some types of human cancers. However, the role of LGR5 in cervical cancer remains unclear. In this study, the expression of LGR5 gradually increases from normal cervix to cervical cancer *in situ* and to cervical cancers as revealed by immunohistochemistry and western blot analyses. Through knocking down or overexpressing LGR5 in SiHa and HeLa cells, the expression level of LGR5 was found to be positively related to cell proliferation *in vitro* and to tumor formation *in vivo*. Further investigation indicated that LGR5 protein could significantly promote the acceleration of cell cycle. Moreover, the TOP-Flash reporter assay and western blot for β-catenin, cyclinD1, and c-myc proteins, target genes of the Wnt/β-catenin pathway, indicated that LGR5 significantly activated Wnt/β-catenin signaling. Additionally, the blockage of Wnt/β-catenin pathway resulted in a significant inhibition of cell proliferation induced by LGR5. Taken together, these results demonstrate that LGR5 can promote proliferation and tumor formation in cervical cancer cells by activating the Wnt/β-catenin pathway.

## INTRODUCTION

Cervical cancer is the third most common type of malignant tumor and the fourth leading cause of cancer death among women worldwide[1, 2]. In developing countries, nearly 500,000 of women develop new cases, and approximately 270,000 women die from cervical cancer each year[3]. Although previous studies have demonstrated that infection by high-risk human papillomaviruses is necessary for cervical cancer[4, 5], the mechanism of cervical carcinogenesis remians unclear. Recently, a number of studies have found that several stem cell-related genes are closely associated with tumorigenesis, and it has been demonstrated that SOX2[6], NANOG[7], and KLF4[8] and OCT4[9] play critical roles in cervical carcinogenesis.

LGR5, also known as GPR49, HG38, or FEX, is a member of the G protein-coupled receptor family comprising proteins with seven transmembrane domains. These proteins are structurally similar to glycoprotein hormone receptors, including thyroid-stimulating hormone receptor, follicle-stimulating hormone receptor and luteinizing hormone receptor[10]. LGR5 has a large N-terminal extracellular domain containing 17 leucine-rich repeats that are important for interaction with their glycoprotein ligands[11, 12]. LGR5 has been identified as a novel marker of adult stem cells in the small intestine and hair follicles[13, 14] and is widely expressed in spinal cord, breast, hair follicles, and brain tissues[11]. LGR5 also plays an important role during embryogenesis.

In recent years, many studies have revealed that LGR5 is overexpressed in various types of tumors, including colorectal cancer[15], ovarian tumor[16], hepatocellular carcinoma[17], basal cell carcinoma[18], and esophageal adenocarcinoma[19]. High LGR5 expression is associated with the initiation, invasion, and metastasis of tumors[20, 21], suggesting the potential role of LGR5 in tumorigenesis. Additionally, LGR5 has been recognized as a cancer stem cell marker for colorectal cancers[22]. As all know, Wnt/β-catenin pathway, as an ancient and highly conserved system, plays critical role in the regulation of stem and cancer stem cells[23], and abnormally activated Wnt/β-catenin pathway is usually associated with tumorigenesis[24]. Moreover, increasing evidence showed that LGR5 also was involved in Wnt/β-catenin pathway in the regulation of stem cells[25, 26]. However, to our knowledge, the role of LGR5 in cervical carcinogenesis remains unclear. Therefore, we hypothesized that LGR5 might also contribute to cervical carcinogenesis, and in this study, we investigated the role of LGR5 in cervical cancer. Our findings showed that LGR5 was progressively expressed in cervical carcinogenesis, and LGR5 expression promoted the proliferation and tumor formation of cervical cancer cells by potentiating the Wnt/β-catenin pathway, indicating that LGR5 may be a potential therapeutic target in cervical cancer.

## RESULTS

### LGR5 expression in human normal cervix and cervical cancerous lesions

**Figure 1:**
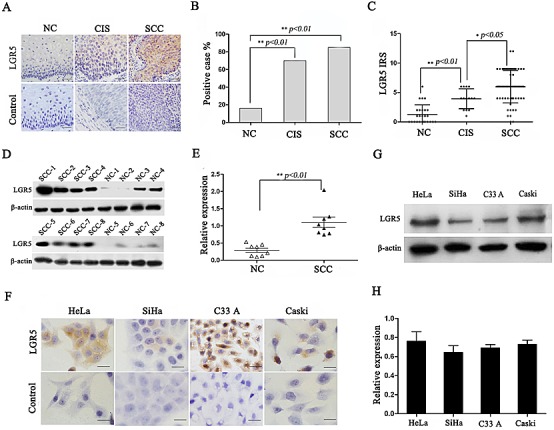
LGR5 expression in cervical cancer tissues and cell lines (A) Immunohistochemical staining showing LGR5 expression in normal cervix, cancer *in situ*, and cervical cancer, scale bar, 50 μm. (B) Bar graph showing the LGR5-positive percentage of normal cervix, cancer *in situ*, and cervical cancer, Chi-Square test was performed. (C) The immunoreactivity score of LGR5 staining in normal cervix, cancer *in situ*, and cervical cancer, and One-Way ANOVA was performed. (D) Western blot analysis of LGR5 expression in normal cervix and cervical cancer tissues; representative blots are shown. (E) Quantitative analysis of LGR5 expression in normal cervix and cervical cancer; β-actin was used as a loading control, student's t-test was carried out. (F) The expression of LGR5 in HeLa, SiHa, C33A, and Caski cells was measured by western blot. (G) Immunocytochemical staining for LGR5 expression in HeLa, SiHa, C33A, and Caski cells, scale bar, 50 μm. (H) The relative expression of LGR5 in HeLa, SiHa, C33A, and Caski cells was calculated based on western blot analyses. NC: Normal cervix; CIS: Cancer in situ; SCC: Squamous cervical cancer. Values are shown as the mean±SD, * *p<0.05*, ** *p<0.01*.

To understand whether LGR5 is involved in cervical carcinogenesis, endogenous LGR5 expression was examined in human normal cervix, cervical cancer in situ, invasive cervical cancer, and cervical cancer cell lines. Representative cytoplasmic LGR5 staining using immunohistochemistry is shown in Fig. 1A. The positive LGR5 expression rates were 17% (5/30) in normal cervix, 65% (11/17) in cancer in situ, and 84% (54/64) in cervical cancers (Fig. 1B, p<0.01). Accordingly, analysis of the IRS of LGR5 also revealed that LGR5 expression is significantly increased from normal cervix to cervical cancer in situ and finally to cervical cancers (p<0.01, Fig. 1C). To further confirm the LGR5 expression results in cervical carcinogenesis, a western blot assay was used to analyze 8 samples of randomly selected normal cervix and cervical cancer tissues. A representative blot is shown in Fig. 1D, and the relative quantitative expression of LGR5 is summarized in Fig. 1E. LGR5 expression in cervical cancer was approximately twofold greater than that in normal cervix (p<0.05). Furthermore, the LGR5 protein was found to be highly expressed in the cytoplasm of all cervical cancer cell lines (HeLa, SiHa, C33A, and Caski) by immunohistochemistry (Fig. 1F) and western blot assay (Fig. 1G and 1H). All these results indicated that LGR5 may function as an oncogene involved in the development and progression of cervical carcinogenesis.

### LGR5 promotes the tumor formation of cervical cancer cells *in vivo*

**Figure 2:**
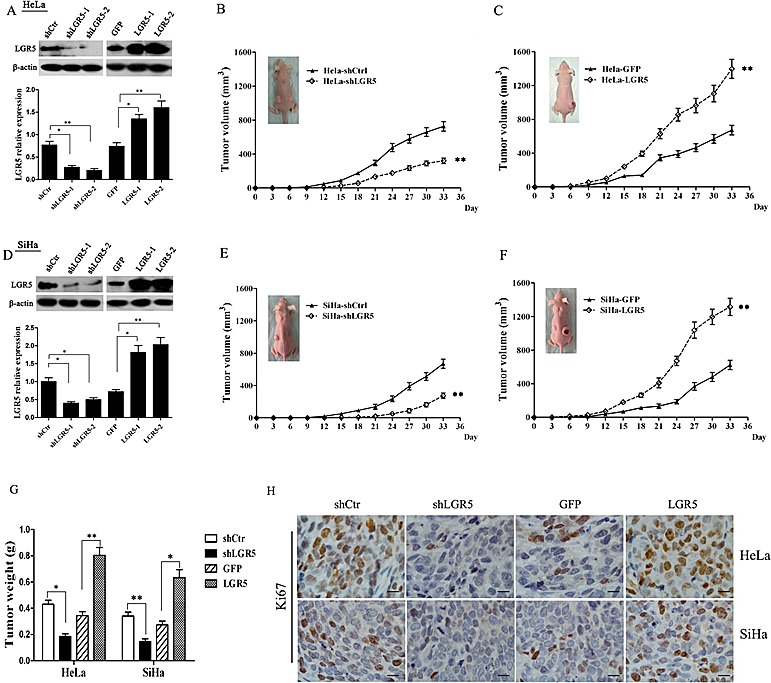
LGR5 promotes the growth of cervical cancer xenografts *in vivo* (A) A western blot assay was used to characterize the expression of LGR5 in LGR5-knockdown and -overexpressing HeLa cells. (B) The tumor growth curve of LGR5-knockdown HeLa cells injected into female nude mice is shown. (C) The tumor growth curve of LGR5-overexpressing HeLa cells injected into female nude mice is shown. (D) A western blot assay was used to characterize the expression of LGR5 in LGR5-knockdown or -overexpressing SiHa cells. (E) The tumor growth curve of LGR5-knockdown SiHa cells injected into female nude mice is shown. (F) The tumor growth curve of LGR5-overexpressing SiHa cells injected into female nude mice is shown. (G) At the end of the experiment, the tumor tissues were dissected, and the weights were measured. (H) Immunohistochemical staining for Ki67 in tumor tissues, scale bar, 10 μm. shLGR5: shRNA for LGR5; shCtr: shRNA for control; GFP: green fluorescent protein. Values are shown as the mean±SD. * *p<0.05*, ** *p<0.01 vs.* control using One-Way ANOVA.

To assess the effects of LGR5 expression in tumor formation, stable LGR5-knockdown cells (HeLa-shLGR5 and SiHa-shLGR5) and stable LGR5-overexpressing cells (SiHa-LGR5 and HeLa-LGR5) were established by shRNA and plasmid transfection. The expression of LGR5 in the knocked down and overexpressing HeLa and SiHa cells by western blot analysis are shown and quantitatively summarized in Fig. 2A and 2D; the levels were found to be effectively down-regulated or overexpressed, respectively. Nude mice were injected subcutaneously with these LGR5-modulated cervical cancer cells, and the growth of tumors was monitored in terms of tumor volume every three days. At the termination of the experiment, the mice were sacrificed, and the tumors were excised; the wet weights of the tumors were recorded.

As shown in Fig. 2B, palpable tumors formed by the shLGR5 HeLa cells were observed at 15 days after inoculation, but tumors formed by the shControl HeLa cells were found at day 12. The tumors formed by the shLGR5 HeLa cells grew much slower than those formed by the shControl HeLa cells (Fig. 2B, p<0.01). In addition, the weights of the tumors formed by the shLGR5 HeLa cells (0.19±0.02 g) were much reduced compared to the shControl HeLa cells (0.43±0.03 g) (Fig. 2G, p<0.05). All these data indicated that down-regulated LGR5 may attenuate the tumor initiation and progression of HeLa cells. Although there was no significant difference between the LGR5-overexpressing HeLa cells and GFP control cells with regard to palpable tumor formation (both for 9 days), tumor progression by the LGR5-overexpressing cells was much faster than that by the HeLa-GFP control cells (Fig. 2C, p<0.01). The weights of the tumor due to the LGR5-overexpressing cells (0.80±0.05 g) were also much heavier than those due to the HeLa-GFP control cells (0.35±0.03 g) (Fig. 2G, p<0.01). These data suggested that overexpressing LGR5 may enhance the tumor progression of HeLa cells. Similarly, palpable tumor formation required 18 days for the shLGR5 SiHa cells but only 12 days for the shControl SiHa cells (Fig. 2E). Moreover, the tumors formed by the shLGR5 SiHa cells grew much slower than those formed by the shControl SiHa cells (Fig. 2E, p<0.01), and the weights of the tumors formed by the shLGR5 SiHa cells (0.15±0.02 g) were much less than those formed by the shControl SiHa cells (0.34±0.03 g) (Fig. 2G, p<0.01). Therefore, down-regulating LGR5 could attenuate tumor initiation and tumor progression in SiHa cells. Furthermore, the tumors formed by the LGR5-overexpressing SiHa cells grew much faster (Fig 2F, p<0.01) and were much heavier (Fig. 2G, p<0.05) than those formed by the SiHa-GFP cells. These results indicate that LGR5 can promote the tumor growth of cervical cancer cells.

To determine whether LGR5 enhances the tumor progression of cervical cancer by promoting cell proliferation, the expression of Ki67, a well-known cell proliferation marker, was examined in the tumor xenografts tissues by immunohistochemical staining. As shown in Fig. 2H, the expression of Ki67 in the tumor tissues formed by the shLGR5 HeLa and SiHa cells was decreased compared with the shControl cells. In contrast, many more Ki67 positive cells were found in the tumor tissues formed by the LGR5-overexpressing HeLa and SiHa cells than in those formed by the GFP HeLa and SiHa cells. All these data suggest that LGR5 most likely enhances the tumor progression of cervical cancer cells by promoting cell proliferation.

### LGR5 promotes the proliferation of cervical cancer cells by accelerating the cell cycle

**Figure 3:**
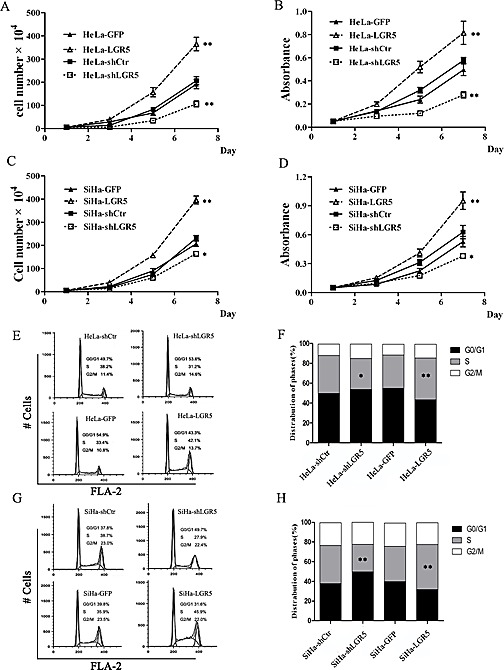
LGR5 promotes the proliferation of cervical cancer cells by accelerating the cell cycle (A) The effect of LGR5 on the proliferation of HeLa cells. (B) The effect of LGR5 on the viability of HeLa cells. (C) The effect of LGR5 on the proliferation of SiHa cells. (D) The effect of LGR5 on the viability of SiHa cells. (E) The cell cycle of LGR5-modulated HeLa cells was analyzed by flow cytometry. (F) The effect of LGR5 on the cell cycle of HeLa cells. (G) The cell cycle of LGR5-modulated SiHa cells was analyzed by flow cytometry. (H) The effect of LGR5 on the cell cycle of SiHa cells. shLGR5: shRNA for LGR5, shCtr: shRNA for control, GFP: green fluorescent protein. Values are shown as the mean±SD collected from three experiments in duplicate. One-Way ANOVA was performed and * *p<0.05*, ** *p<0.01 vs.* control.

To further uncover the potential mechanism underlying tumor growth promotion by LGR5, a cell growth curve assay and the MTT assay were performed *in vitro*. The growth curve (Fig. 3A and 3C) revealed that LGR5 knockdown in both HeLa (Fig. 3A) and SiHa (Fig. 3C) cells resulted in a significant growth inhibition. However, LGR5 overexpression markedly promoted cell growth (Fig. 3A and 3C, p<0.01). Furthermore, MTT assays with HeLa and SiHa cells confirmed that LGR5 knockdown resulted in a significant decrease in cell viability and that LGR5 overexpression led to a marked increase in cell viability (Fig. 3B and 3D, p<0.01). These results demonstrate that LGR5 can promote the proliferation of cervical cancer cells.

Because cell proliferation changes usually involve modulation of the cell cycle, the HeLa and SiHa cell cycle was analyzed by flow cytometry to examine whether LGR5 promotes cell proliferation by affecting the cell cycle. A representative histogram is shown in Fig. 3E and 3G, and the results are summarized in Fig. 3F and 3H. LGR5 knockdown resulted in a marked decrease in the percentage of both HeLa (Fig. 3F) and SiHa (Fig. 3H) cells in S phase. Conversely, LGR5 overexpression significantly increased the S-phase percentage both cell types (Fig. 3F and 3H). Collectively, these results suggest that LGR5 promotes the tumor growth of cervical cancer cells, possibly by accelerating the cell cycle.

### LGR5 potentiates the Wnt/β-catenin pathway in cervical carcinogenesis

It has been reported that LGR5 regulates Wnt/β-catenin signaling by associating with R-spondin[25, 31] and enhances cell proliferation in intestinal epithelium and Ewing sarcoma[32, 33]. However, there are no reports identifying whether LGR5 is able to enhance the proliferation and tumor formation of cervical cancer cells by activating Wnt/β-catenin signaling.

**Figure 4:**
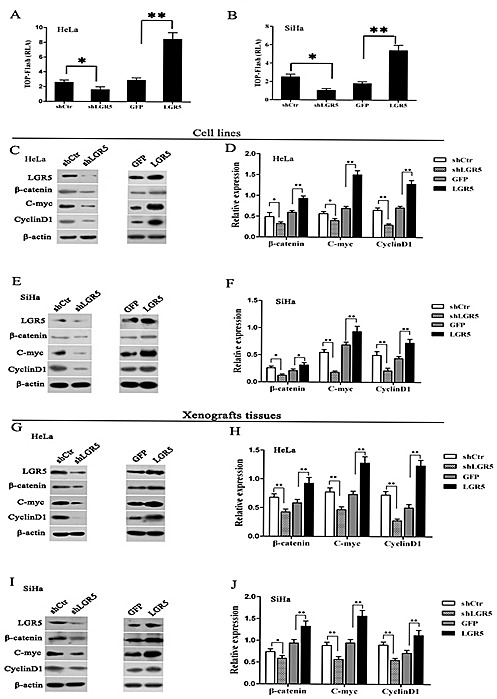
LGR5 enhances the activity of the Wnt/β-catenin pathway (A, B) LGR5-modulated HeLa and SiHa cells were transfected with the TOP-Flash reporter plasmid, and the reporter activities were determined 48 h after transfection by a luciferase assay. LGR5 significantly increased the reporter activity in HeLa and SiHa cells. (C) The expression of β-catenin, c-myc, and cyclinD1 in LGR5-modulated HeLa cells was measured by a western blot assay. (D) The quantitative analysis of β-catenin, c-myc, and cyclinD1 expression in LGR5-modulated HeLa cells. (E) Representative blots showing the expression of β-catenin, c-myc, and cyclinD1 in LGR5-modulated SiHa cells; (F) The quantitative analysis of these proteins is shown. (G, I) The expression of β-catenin, c-myc, and cyclinD1 in tumor xenografts was measured by western blot; (H, J) The relative expression of these proteins was normalized to β-actin. Values are shown as the mean±SD of three independent experiments. * p<0.05, ** p<0.01 vs. control using One-Way ANOVA.

**Figure 5:**
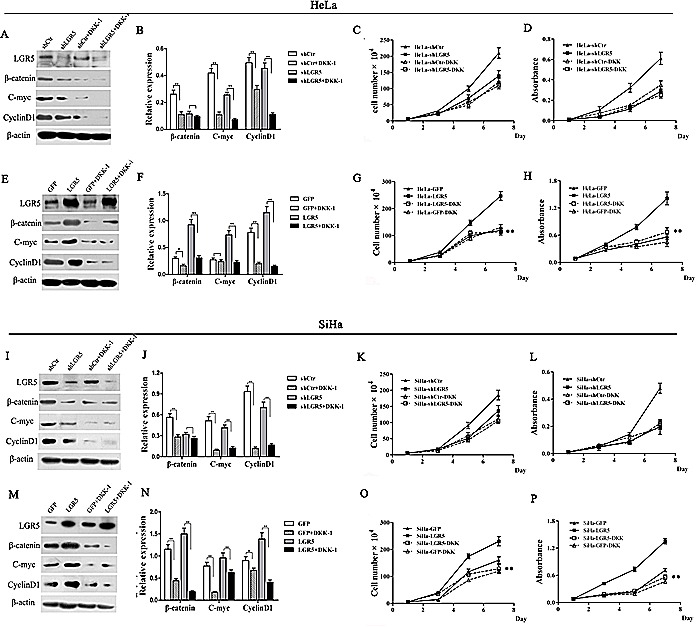
DKK-1 treatment attenuates the increasing proliferation of cervical cancer cells induced by LGR5 LGR5-modulated HeLa and SiHa cells were treated with the Wnt inhibitor DKK-1 (200 ng·ml^−1^), and the expression of β-catenin, c-myc, and cyclinD1was measured by western blot. (A) The representative blot and (B) quantitative analysis of β-catenin, c-myc, and cyclinD1 expression were shown. (C) The effect of DKK-1 on the proliferation of LGR5-knockdown HeLa cells was evaluated by cell counting. (D) The effect of DKK-1 on the viability of LGR5-knockdown HeLa cells was measured by the MTT assay. (E) The representative blot and (F) quantitative analysis of β-catenin, c-myc, and cyclinD1 expression were shown. The effect of DKK-1 on the (G) proliferation and (H) viability, respectively, of LGR5-overexpressing HeLa cells was evaluated. (I, J) The expression of β-catenin, c-myc, and cyclinD1 was measured. (K, L) The effect of DKK-1 on the proliferation and viability, respectively, of LGR5-knockdown SiHa cells was evaluated. (M, N) The expression of β-catenin, c-myc, and cyclinD1 was measured. (O, P) The effect of DKK-1 on the proliferation and viability, respectively, of LGR5-overexpressing SiHa cells was evaluated. Values are shown as the mean±SD of three experiments in duplicate. * *p<0.05*, ** *p<0.01 vs.* control using One-Way ANOVA.

The TOP-Flash reporter assay is a canonical experiment for the detection of Wnt/β-catenin signaling activity. Therefore, the TOP-Flash reporter assay was used to detect the activity of Wnt/β-catenin signaling in cervical cancer cell lines (Fig. 4A andB) in which LGR5 was overexpressed or down-regulated. The results show that LGR5 knockdown resulted in a significant inhibition of TOP-Flash reporter activity in HeLa cells (p<0.05), whereas LGR5 overexpression significantly increased the TOP-Flash reporter activity in HeLa cells by 2- or 3-fold compared with the control (p<0.01, Fig. 4A). Similar results were also observed in LGR5-knockdown and -overexpressing SiHa cells (Fig. 5B). All these results indicate that LGR5 expression is positively related to the activity of the Wnt/β-catenin pathway in cervical cancer cells.

β-catenin is a crucial signaling molecule, and cyclinD1 and c-myc are important target genes of the Wnt/β-catenin pathway. Therefore, the expression of β-catenin, cyclinD1, and c-myc proteins was measured by a western blot assay in LGR5-down-regulated and -overexpressing HeLa and SiHa cells. Representative blots for HeLa and SiHa cells are shown in Fig. 4C and 4E, respectively, and the relative expression of these proteins was further calculated by normalization to β-actin expression, as summarized in Fig. 4D and 4F, respectively. The results show that the expression of β-catenin, cyclinD1, and c-myc proteins in LGR5-knocked down HeLa cells was significantly decreased compared with the control cells. In contrast, the expression of these proteins in LGR5-overexpressing HeLa cells was significantly increased compared with the control cells. Similar results were observed in LGR5-knockdown or -overexpressing SiHa cells. All these results demonstrate that LGR5 expression is positively associated with the activity and expression of key molecules of the Wnt/β-catenin pathway in cervical cancer cells.

To test whether the expression of LGR5 is also associated with Wnt/β-catenin signaling *in vivo*, the expression of the β-catenin, cyclinD1, and c-myc proteins was examined by a western blot assay in the xenograft tumor tissues formed by LGR5-down-regulated and -overexpressing cervical cancer cells. Representative blots are shown in Fig. 4G and 4I, respectively, and the relative expression of these proteins was further calculated by normalization to β-actin expression, as summarized in Fig. 4H and 4J, respectively. The expression of β-catenin, cyclinD1, and c-myc in the tumor tissues formed by the LGR5-knockdown HeLa and SiHa cells were markedly reduced compared with their controls (p<0.01), whereas expression in the tissues formed by the LGR5-overexpressing HeLa and SiHa cells were significantly increased compared with their controls (p<0.01). All these results suggest that the LGR5-promoted proliferation and tumor formation of cervical cancer cells *in vivo* is possibly mediated by potentiating the Wnt/β-catenin pathway.

### Blockage of the Wnt/β-catenin pathway by DKK-1 attenuates the cell proliferation mediated by LGR5 expression

Dickkopf-1 (DKK-1) is an inhibitor that can antagonize the Wnt/β-catenin pathway by binding to LRP6[34]. To further confirm that the Wnt/β-catenin pathway is the pathway by which LGR5 promotes the proliferation of cervical cancer cells, DKK-1 was used to block Wnt/β-catenin pathway in LGR5-modulated HeLa and SiHa cells.

The protein levels of cyclinD1 and c-myc in the DKK-1-treated, LGR5-modulated HeLa and SiHa cells were significantly decreased compared to those in the cells without DKK-1 treatment, regardless of whether LGR5 was knocked down or overexpressed in the cells (Fig. 5A-5N). This result suggests that DKK-1 treatment eliminated the potentiation of the Wnt/β-catenin pathway by LGR5, indicating that LGR5 indeed influences the activity of the Wnt/β-catenin pathway and that the site of DKK-1 action is located downstream of LGR5. Treatment with DKK-1 also resulted in a significant reduction in the expression of β-catenin, a key signal molecule of the Wnt/β-catenin pathway, in the LGR5-overexpressing cells. There was no significant change in β-catenin expression in the LGR5-knockdown SiHa and HeLa cells with and without DKK1 treatment, though β-catenin expression was slightly decreased in the DKK-1-treated cells (Fig. 5A-5N). This finding may be attributed to the low level of β-catenin expression in the LGR5-knockdown cells and confirms the role of LGR5 in modulating the activity of the Wnt/β-catenin pathway.

Consistent with the observations above, DKK-1 treatment resulted in a significant inhibition in cell proliferation and viability in the LGR5-overexpressing HeLa and SiHa cells (p<0.01, Fig. 5G, 5H, 5O, 5P), indicating that DKK-1 can arrest the cell proliferation and viability induced by LGR5. However, there was no obvious change observed in the LGR5-knockdown cells treated with DKK-1 (Fig. 5 C, D, K, and L). We speculate that because the knockdown of LGR5 already resulted in low LGR5 protein expression, low Wnt/β-catenin pathway activity, and low proliferative ability, DKK-1 treatment could not make a significant change, even though this inhibitor also affects the Wnt/β-catenin pathway. Taken together, these results demonstrate that the LGR5-mediated promotion of cervical cancer cell proliferation is mediated by the Wnt/β-catenin pathway.

### Correlation among LGR5, β-catenin, cyclinD1, and c-myc expression in cervical cancer

**Figure 6:**
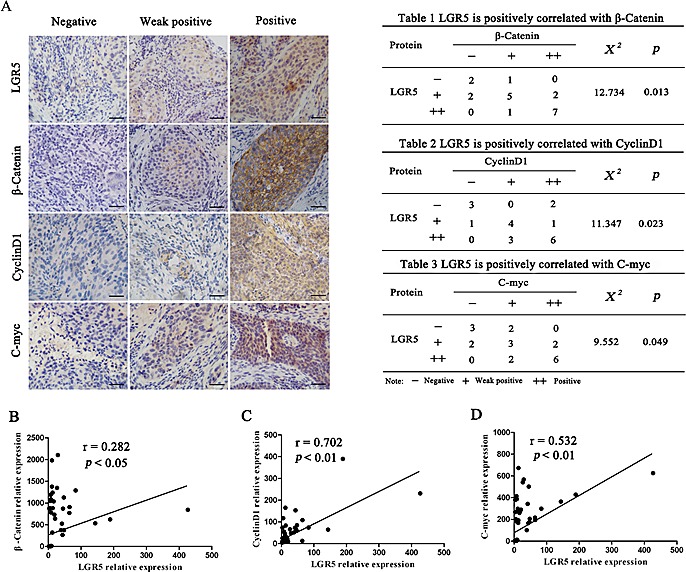
LGR5 expression is positively correlated with the expression of Wnt signaling-related proteins in human cervical cancer tissues (A) Twenty cervical cancer specimens were analyzed by immunohistochemical staining, and the representative expression of LGR5, β-catenin, c-myc, and cyclinD1 is shown, scale bar, 50 μm. (B, C, D) Correlation of LGR5 and β-catenin, c-myc, and cyclinD1 mRNA expression, respectively, in human cervical cancer from dataset GSE5787 (n = 82) was analyzed. Correlation analysis was performed using pearson Chi-Square test.

To elucidate the clinical relevance of LGR5 and Wnt/β-catenin signaling in cervical cancer tissues, we examined the association between endogenous LGR5 and β-catenin, cyclinD1, and c-myc expression in human cervical cancer by immunohistochemical staining (Fig. 6A) and reanalyzing cDNA microarray databases from an established human cervical cancer study (Fig. 6B, 6C, 6D). We found that LGR5 expression was positively correlated with β-catenin, cyclinD1, and c-myc expression in randomly selected cervical cancer sections (Tables 1-3). In addition, an analysis of the GSE5787 microarray datasets for 82 cervical cancer patients also showed that LGR5 expression had a significant correlation with the expression of these proteins. Therefore, these data support the notion that LGR5 is associated with increased activity of the Wnt/β-catenin pathway in cervical cancer.

## DISCUSSION

LGR5, a member of the G-protein-coupled receptor family of proteins, has been identified as a stem cell marker of the small intestine and colon^33^, as well as hair follicles[35, 36]. In recent years, the overexpression of LGR5 has been observed in many types of cancers, including hepatocellular carcinoma[17], colorectal cancer[37], ovarian cancer[38], and basal cell carcinoma[18], suggesting that LGR5 may play an important role in tumorigenesis. However, to our knowledge, the role of LGR5 in cervical cancer remains unclear. We for the first time found that the expression of LGR5 was gradually increased from normal cervix (17%) to cancer in situ (65%) and invasive cervical cancer (84%), suggesting that LGR5 may function to promote the development and progression of cervical cancers (Fig. 1), as in other types of cancers[17, 18, 39].

Subsequently, through shRNA knockdown or stable plasmid transfection, the LGR5 protein level was found to be positively related to the proliferation of cervical cancer cells (Fig. 3). Tumor xenograft experiments in nude mice indicated that LGR5 significantly promoted tumor growth *in vivo* (Fig. 2). Moreover, immunostaining assays revealed that the tumor tissues formed by LGR5-overexpressing cells had much stronger Ki67 expression, suggesting that LGR5 promoted the tumor formation of cervical cancer cells by accelerating cell proliferation (Fig. 2). Furthermore, a cell cycle analysis by FACS revealed that increased LGR5 expression resulted in a significant increase in the percentage of cells in S phase and a concomitant decrease in the percentage of G_0_/G_1_ phase cells (Fig. 3), suggesting that LGR5 accelerates the cell cycle in cervical cancer cells. All of our findings together indicate that LGR5 functions to promote the development and progression of cervical cancer, consistent with previous reports in basal cell carcinoma[18] and malignant glioma[40]. However, Walker F et al reported an inhibitory effect of LGR5 on cell proliferation in colorectal cancer[41], suggesting that LGR5 may have different impacts on different types of carcinomas.

Recently, LGR5 was found to potentiate Wnt/β-catenin signaling in HEK293T cells[25] and in Ewing sarcoma[33], and LGR5 was found to function as a receptor of R-spondins to enhance Wnt-induced LRP phosphorylation[25, 31, 42] and then activate Wnt/β-catenin signaling. In the present study, LGR5 was found not only to significantly enhance the activity of the TOP-Flash reporter in HeLa and SiHa cells but also to increase the expression of target genes of the Wnt/β-catenin pathway in HeLa and SiHa cells and xenograft tumors (Fig. 4). These results suggest that LGR5 enhances the activity of the Wnt/β-catenin pathway in cervical cancer cells. In addition, a correlation analysis from our immunochemical staining data from cervical cancer tissues as well as cervical cancer cDNA microarray datasets (GSE5787) indicated that LGR5 expression is positively correlated with the expression of target genes of the Wnt/β-catenin pathway in human cervical cancer tissues (Fig. 6), which indirectly supports our proposal that LGR5 may potentiate the Wnt/β-catenin pathway in cervical cancers.

An increasing number of studies have demonstrated that LGR5 promotes tumorigenesis, accelerates cell proliferation, and activates the Wnt/β-catenin pathway [43]. However, it remains inconclusive whether LGR5 promotes cell proliferation and tumorigenesis by activating the Wnt/β-catenin pathway. In the present study, blockage with DKK-1, an inhibitor of Wnt/β-catenin signaling, resulted in a significant inhibition of the cervical cancer cell proliferation induced by LGR5 (Fig. 5), suggesting that LGR5 promotes cervical cancer cell proliferation via activation of the Wnt/β-catenin pathway.

**Figure 7:**
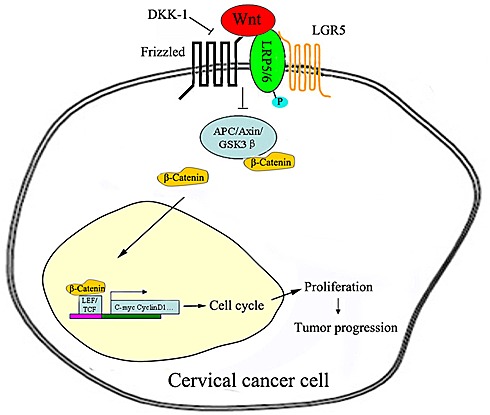
Proposed model of the LGR5-mediated promotion of cervical cancer growth via the Wnt/β-catenin signaling pathway Once activated, the LGR5 protein recruits the LRP-Frizzled receptor complex, which binds to Wnt ligands and reinforces Wnt signaling following the phosphorylation of LRP. A series of steps ensue, including the accumulation of β-catenin, which is translocated to the nucleus and, together with the TCF/LEF family of transcription factors, increases related gene expression, ultimately leading to the progression of cervical cancer.

In summary, our study demonstrates that LGR5 promotes cell proliferation and tumor formation in cervical cancer cells by activating the Wnt/β-catenin pathway. Therefore, we propose a hypothesis in which LGR5 potentiates Wnt/β-catenin signaling, accelerates the cell cycle, and promotes cell proliferation, eventually leading to the progression of cervical cancer (Fig. 7). Furthermore, the Wnt/β-catenin pathway has been found to play an essential role in the self-renewal and maintenance of stem cells[44, 45]. Therefore, further investigation is necessary to clarify whether LGR5 enhances the tumor formation of cervical cancer cells through the regulation of cervical cancer stem cells.

## MATERIALS AND METHODS

### Cell lines and cell culture

Human cervical cancer cell lines (HeLa, SiHa, C33A, Caski) and a human colon adenocarcinoma cell line (SW620) were purchased from American Type Culture Collection (ATCC, Rockville, MD, USA). The HeLa, SiHa, C33A, and SW620 cells were cultured in Dulbecco's Modified Eagle's Medium (DMEM, Sigma-Aldrich, St Louis, MO, USA), and the Caski cells were cultured in RPMI1640 (Sigma-Aldrich, St Louis, MO, USA); culturing was carried out at 37°C with 5% CO_2_. All media were supplemented with 10% heat-inactivated fetal bovine serum (FBS, Invitrogen, Carlsbad, CA, USA).

### Human tissue samples

A total of 30 normal cervix (NC), 17 cervical cancer in situ (CIS), and 64 squamous cervical cancer (SCC) samples without chemotherapy, immunotherapy, or radiotherapy were obtained via surgery from the First Affiliated Hospital of Medical College, Xi'an Jiaotong University between January 2009 and December 2011. The study was approved by the Ethics Committee of the Medical College of Xi'an Jiaotong University, and the patients gave their informed consent before sample collection.

### Immunohistochemistry and immunocytochemistry

The immunohistochemical staining procedure was performed as previously described[27]. Briefly, formalin-fixed and paraffin-embedded tissue sections (4 μm thick) were deparaffinized in xylene and rehydrated through descending concentrations of ethanol. After antigen retrieval was performed by heating in 10 mM citrate buffer (pH 6.0) for 2 min, the sections were treated with 3% hydrogen peroxide to block endogenous peroxidase. Subsequently, the sections were incubated with a primary antibody overnight at 4°C. A horseradish peroxidase-conjugated secondary antibody was added for 30 min at room temperature, followed by 3,3′-diaminobenzidine development. The sections were counterstained with hematoxylin. As a negative control, the primary antibody was replaced with PBS.

LGR5 staining was classified into two categories, negative and positive expression, based on the percentage of positive cells and the staining intensity[28]. The staining intensity was scored as follows: 0, negative; 1, weak; 2, moderate; 3, strong. The percentage of positive cells was divided into five rankings: 0, ≤ 5%; 1, 5% to 25%; 2, 25% to50%; 3, 50% to 75%; 4, >75%. The final score was determined by multiplying the intensity score and the quantity score using the following formula: immunoreactivity score(IRS) = intensity score ×quantity score. The expression of LGR5 was defined as positive when the score was >3. All specimens were evaluated by two pathologists in a blinded manner.

For immunocytochemistry, cells were plated on cover slips, fixed with 4% paraformaldehyde for 30 min at room temperature, and permeabilized with 0.2% Triton X-100 for 20 minutes at room temperature. A staining procedure similar to that described above was performed. Antibody against LGR5 was obtained from Abnova (Taipei, Taiwan), other antibodies against Ki67, β-catenin, cyclinD1 and c-myc were obtained from Santa Cruz Biotechology (Santa Cruz, CA, USA).

### Vector construction and transfection

Human full-length LGR5 cDNA was amplified by reverse transcription polymerase chain reaction using mRNA extracted from SW620 cells. The primer sequences were designed as follows:

F5′-CTTCTCGAGCTACTTCGGGCACCATGG AC-3′;

R5′-GCGGGTACCTTAGAGACATGGGACAAA TG-3′.

The LGR5 DNA fragment was subsequently cloned into the XhoI and SmaI sites of the pCAG-AcGFP vector (Clontech, Mountain View, CA, USA) to generate the pCAG-AcGFP-LGR5 recombinant plasmid. The small interfering RNA expression vector that expresses LGR5-specific short hairpin RNA (shRNA) was purchased from GenePharma Co., Ltd (Shanghai, China). The LGR5 overexpression and shRNA vectors were transfected into SiHa and HeLa cells using the Lipofectamine 2000 reagent (Invitrogen, Carlsbad, CA, USA) according to the manufacturer's protocol. The transfected cells were treated with G418 (Calbiochem, La Jolla, CA, USA) for 3 weeks, and drug-resistant colonies were collected, expanded, and identified.

### Western blot analysis

A western blot analysis was carried out as previously described[29]. In brief, the lysates from cells and fresh tissues were separated by SDS-PAGE and transferred onto PVDF membranes. After blocking with 5% fat-free milk, the membranes were incubated with a primary antibody against human LGR5 (1:200 dilution; Abnova, Taipei, Taiwan), β-actin (1:1000 dilution; Santa Cruz, CA, USA), β-catenin (1:500 dilution; Santa Cruz), c-myc (1:500 dilution, Santa Cruz), or cyclinD1(1:500 dilution, Santa Cruz) at 4°C overnight, followed by secondary incubation using a horseradish peroxidase-conjugated anti-rabbit or anti-mouse IgG (Thermo Fisher Scientific, New York, NY, USA). The proteins were visualized with an enhanced chemiluminescence reagent (Millipore, Billerica, MA, USA) after exposure to X-ray films. The LGR5 western blot results were normalized to those of β-actin blotting for quantification.

### Cell proliferation and viability assays

Cells were seeded in 6-well plates at the concentration of 5×10^4^ cells/well and incubated as described above. The cells were harvested and counted at 1, 3, 5, and 7 days using a hemocytometer, and cell proliferation was assessed by cell growth curves. To test cell viability, 1×10^3^ cells were cultured in 96-well plates for 7 days, and MTT assays were performed according to the manufacturer's protocol. All experiments were performed independently in triplicate.

### Flow cytometry

For cell cycle analysis, 1×10^6^ cells were harvested and washed twice with cold PBS, followed by fixation with ice-cold 70% ethanol overnight at 4°C. After washing twice with PBS, the cells were incubated with propidium iodide (Sigma-Aldrich, St. Louis, MO, USA) and RNaseA for 30 min at room temperature. The cells were then analyzed using a FACS Calibur (BD Biosciences, San Jose, CA, USA) with CellQuest software.

### Tumor xenograft experiment

Female BALB/c-nude mice (6-7 weeks old) were obtained from Slac Laboratory Animal Co., Ltd (Shanghai, China) and housed in an SFP room that was maintained at a constant temperature (22°C-25°C) and humidity (40-50%). Tumor cells (1×10^6^) were injected into the subcutis on the dorsum of each mouse. The tumor size was measured using a vernier caliper every week, and the volume was calculated with the following formula: V= (length×width^2^)/2. At the termination of the experiment, the tumors mass was harvested, weighed, and stored for immunostaining or protein extract. All animals received humane treatment in accordance with institutional policies, and all studies were approved by the Animal Care and Use Committee of the Medical School of Xi'an Jiaotong University.

### Reporter assay

TOP-Flash assays were performed as previously described[30]. In brief, TOP-Flash reporter and pTK-RL plasmids were transiently co-transfected into tumor cells with Lipofectamine 2000 (Invitrogen, Carlsbad, CA, USA) in 24-well plates, and the activity of both firefly and Renilla luciferase reporters was determined at 48 hours after transfection using the Dual Luciferase Assay kit (Promega, Madison, WI, USA) according to the manufacturer's instructions. The TOP-Flash reporter activity is presented as the relative ratio of firefly luciferase activity to Renilla luciferase activity. All experiments were performed three times with triplicate replicates.

### Statistical analysis

A statistical analysis was performed using SPSS software version 16.0 (SPSS Inc., Chicago, IL, USA). For comparison among the groups, a Chi-Square test or an One-Way ANOVA followed by a post hoc Tukey test was performed, and p < 0.05 was defined as statistically significant. The data are shown as the means±SD.
